# Screening speech disorders in progressive neurological diseases via long-term average spectrum

**DOI:** 10.1007/s00702-026-03176-2

**Published:** 2026-05-14

**Authors:** Jan Svihlik, Jan Rusz

**Affiliations:** 1 Department of Circuit Theory, Faculty of Electrical Engineering, Czech Technical University in Prague, Technická 2, Prague 6, 160 00 Czechia; 2 Department of Mathematics, Informatics, and Cybernetics, Faculty of Chemical Engineering, University of Chemistry and Technology, Prague 6, Czechia; 3 Department of Neurology, Centre of Clinical Neuroscience, First Faculty of Medicine, Charles University and General University Hospital, Prague, Czechia

**Keywords:** Dysarthria, Speech disorder, Long-term average spectrum, Acoustic analysis

## Abstract

The long-term averaged spectrum (LTAS) may provide a universal method for capturing distinct patterns of dysarthria. This study aimed to evaluate the sensitivity of LTAS descriptors in a broad range of neurological diseases and various types and severities of dysarthria. Four spectral moments of spectral mean, spectral standard deviation, spectral skewness and spectral kurtosis based on LTAS were computed for reading passage collected from 461 speakers, including 306 healthy controls and 155 neurological patients secondary to Parkinson’s disease (PD), progressive supranuclear palsy, multiple system atrophy (MSA), Huntington’s disease, essential tremor, cerebellar ataxia (CA), multiple sclerosis (MS), and amyotrophic lateral sclerosis. Compared to controls, the spectral mean was significantly lower in PD and MS while elevated in CA. Significantly changed LTAS features were observed only in hypokinetic dysarthria and in mixed dysarthrias manifesting hypokinetic elements. Although LTAS features differed between controls and patients with varying degrees of dysarthria, there was no progressive increase in dysarthria severity. Our findings suggest that LTAS-based speech analysis may provide valuable cues to aid differential diagnosis among neurological diseases with overlapping clinical features. LTAS appears more informative when applied to specific diseases than to pooled dysarthria types arising from diverse neurological etiologies.

**Supplementary Information:**

The online version contains supplementary material available at https://doi.org/10.1007/s00702-026-03176-2.

## Introduction

Speech is one of the most complex quantitative markers of motor function and is often affected already in the prodromal stages of many progressive neurological diseases (Rusz et al. 2024). Different neurological disorders give rise to distinct patterns of dysarthric alterations reflecting their underlying pathophysiology, which are broadly classified into the principal dysarthria types: hypokinetic, hyperkinetic, spastic, ataxic, and flaccid (Duffy 2020). In certain conditions, such as Parkinson’s disease (PD), in which most patients develop pure hypokinetic dysarthria (Ho et al. 1998), there is a clear correspondence between disease type and dysarthria classification. Yet, several neurological diseases manifest multiple dysarthria subtypes due to more than one component of the motor system being affected. For instance, atypical parkinsonian syndromes, such as multiple system atrophy (MSA) and progressive supranuclear palsy (PSP), are typically characterized by mixed dysarthria, combining hypokinetic, spastic, and ataxic components (Kluin et al. 1996);(Kluin et al. 1993).

Therefore, the systematic investigation of speech disorders may yield sensitive, easily obtainable biomarkers of disease. For instance, speech can offer valuable clues for the early differential diagnosis of neurological diseases with overlapping clinical features (Duffy 2020; Illner et al. 2024). Also, identifying specific speech biomarkers associated with pallidal, subthalamic, cerebellar, or pyramidal involvement could provide valuable insights for managing treatment-related side effects, such as those arising from deep brain stimulation (Fabbri et al. 2021). However, achieving this requires objective speech biomarkers capable of distinguishing among neurological diseases and their associated dysarthria subtypes. Although the introduction of the Mayo perceptual classification (Darley et al. 1969a);(Darley et al. 1969b) marked a major advance in identifying distinctive dysarthria patterns, its translation into the instrumental acoustic domain remains incomplete. One potential avenue for progress is to investigate the sensitivity of candidate acoustic measures in differentiating among neurological diseases, dysarthria types, and levels of dysarthria severity (Kim et al. 2011).

The long-term averaged spectrum (LTAS), characterized by its spectral moments, may provide a universal method for capturing distinct patterns of dysarthria. This approach applies the discrete Fourier transform to successive windowed segments of the speech signal, then averages the resulting spectra. Physiologically, LTAS has been shown to reflect complex speech changes in voice quality, articulatory decay, articulation rate, and fundamental frequency (Svihlik et al. 2023). In addition, previous studies found relationships between LTAS moments and perceived dysarthria severity (Svihlik et al. 2022; Tjaden et al. 2010), suggesting the potential of LTAS measures to serve as an acoustic proxy of dysarthria severity. However, to date, LTAS has been investigated only in Parkinson’s disease (PD) and multiple sclerosis (MS) (Smith & Goberman 2014; Tjaden et al. 2010). The sensitivity of LTAS moments for distinguishing among various neurological diseases, dysarthria types, and dysarthria severities remains poorly understood.

Therefore, based on a large sample of patients with various progressive neurological diseases secondary to PD, PSP, MSA, Huntington’s disease (HD), essential tremor (ET), cerebellar ataxia (CA), MS, and amyotrophic lateral sclerosis (ALS), we aimed to quantitatively assess the sensitivity of LTAS descriptors to different (i) types of neurological disease, (ii) types of dysarthria, and (iii) severity of dysarthria.

## Methods

### Subjects

Each participant provided written informed consent. This study was approved by the Ethics Committee of the General University Hospital in Prague, Czech Republic, in accordance with the ethical standards established in the 1964 Declaration of Helsinki. Between 2011 and 2021, a total of 461 successive native Czech speakers were recruited for this study. Considering progressive neurodegenerative diseases, 20 patients with PD (10 women, 10 men), 18 with PSP (8 women, 10 men; 13 with Richardson’s syndrome, two with PSP-parkinsonism, and three with PSP-pure akinesia with gait freezing), 20 with MSA (12 women, eight men; 17 with parkinsonian and three with cerebellar variant), 20 with HD (10 women, 10 men), 20 with ET (10 women, 10 men), 18 with CA (eight women, 10 men; 11 with sporadic late-onset CA other than MSA, seven with spinocerebellar ataxia [Type 1, 2, 7, or 8]), 20 with MS (11 women, nine men; 10 with relapsing–remitting MS, five with primary progressive MS, five with secondary progressive MS), and 19 with ALS (14 women, 5 men) were recruited (Table 1).

**Table 1:** Clinical characteristics of the investigated subjects

Disease	Sex	Motor score Mean/SD (range)	Age (years) Mean/SD (range)	Symptom duration (years) Mean/SD (range)	Dysarthria type	Dysarthria severity
PD	F = 10	38.7/14.7^a^	63.5/8.9	1.6/1.3	Hypokinetic (*n* = 20)	Mild (*n* = 13)
	M = 10	(18–70)	(42–79)	(0.3–5.9)		Moderate (*n* = 7) Severe (*n* = 0) Mean severity: 1.35^#^
PSP	F = 8	68.6/29.8^b^	67.1/6.7	4.6/2.5s	Hypokinetic (*n* = 4)	Mild (*n* = 4)
	M = 10	(19–132)	(54–83)	(2.0–11.0)	Hypokinetic-spastic (*n* = 5)	Moderate (*n* = 7)
					Hypokinetic-ataxic (*n* = 3)	Severe (*n* = 7)
					Hypokinetic-spastic-ataxic (*n* = 5) Ataxic (*n* = 1)	Mean severity: 1.8
MSA	F = 12	79.1/21.1^b^	62.0/7.0	4.4/1.8	Hypokinetic (*n* = 3)	Mild (*n* = 1)
	M = 8	(35–115)	(45–73)	(2.0-7.5)	Spastic-ataxic (*n* = 1)	Moderate (*n* = 12)
					Hypokinetic-spastic (*n* = 8)	Severe (*n* = 7)
					Hypokinetic-ataxic (*n* = 3)	Mean severity: 2.30
					Hypokinetic-spastic-ataxic (*n* = 5)	
HD	F = 10	24.8/9.9^c^	53.1/11.0	5.2/3.6	Hyperkinetic (*n* = 20)	Mild (*n* = 1)
	M = 10	(8–42)	(34–69)	(1.0–16.0)		Moderate (*n* = 13)
						Severe (*n* = 6) Mean severity: 2.25
ET	F = 10	17.5/7.6^d^	64.3/11.1	28.9/17.5	Hyperkinetic (*n* = 18)	Mild (*n* = 5)
	M = 10	(6–35)	(40–82)	(3.0–60.0)	Hypokinetic (*n* = 1)	Moderate (*n* = 9)
					Spastic (*n* = 1)	Severe (*n* = 6) Mean severity: 2.05
CA	F = 8	13.9/4.8^e^	54.7/12.6	11.0/8.5	Ataxic (*n* = 5)	Mild (*n* = 5)
	M = 10	(4–24)	(34–72)	(0.5–28.0)	Spastic (*n* = 1)	Moderate (*n* = 7)
					Spastic-ataxic (*n* = 11)	Severe (*n* = 6) Mean severity: 2.06
					Hypokinetic-ataxic (*n* = 1)	
MS	F = 11	4.6/0.8^f^	52.2/10.1	17.8/8.6	Ataxic (*n* = 7)	Mild (*n* = 16)
	M = 9	(4–7)	(33–74)	(6.0–32.0)	Spastic (*n* = 3)	Moderate (*n* = 3)
					Spastic-ataxic (*n* = 10)	Severe (*n* = 1) Mean severity: 1.25^#^
ALS	F = 14	35.6/6.5^g^	62.1/11.1	1.9/1.2	Spastic (*n* = 4)	Mild (*n* = 5)
	M = 5	(22–45)	(37–85)	(0.5-5.0)	Flaccid-spastic (*n* = 16)	Moderate (*n* = 6)
						Severe (*n* = 9) Mean severity: 2.20
Total	F = 83	-	59.8/11.1	9.6/11.8	Hypokinetic (*n* = 28)	Mild (*n* = 50)
	M = 72		(33–85)	(0.3–60.0)	Hyperkinetic (*n* = 38)	Moderate (*n* = 64)
					Ataxic (*n* = 13)	Severe (*n* = 41)
					Spastic (*n* = 9)	Mean severity: 1.94
					Flaccid-spastic (*n* = 15)	
					Spastic-ataxic (*n* = 22)	
					Hypokinetic-spastic (*n* = 13)	
					Hypokinetic-ataxic (*n* = 7)	
					Hypokinetic-spastic-ataxic (*n* = 10)	

^a^ MDS-UPDRS part III total scale, ^b^NNIPPS total scale, ^c^UHDRS total scale, ^d^TETRAS score scale, ^e^SARA total scale, ^f^EDSS total scale, ^g^ ALSFRS-R total scale, ^#^This group was found to have significantly lower disease severity compared to PSP, MSA, HD, ET, CA, and ALS groups with *p* < .01. PD = Parkinson’s disease, PSP = progressive supranuclear palsy, MSA = multiple system atrophy, HD = Huntington’s disease, ET = essential tremor, CA = cerebellar ataxia, MS = multiple sclerosis, ALS = amyotrophic lateral sclerosis

All patients were examined by a neurologist with an experience in movement, demyelinating, or neuromuscular disorders. The diagnosis of PD was established by the Movement Disorders Society clinical diagnostic criteria (Postuma et al. 2015); PSP by the Movement Disorder society diagnostic criteria for PSP (Höglinger et al. 2017); MSA by the consensus diagnostic criteria for MSA (Gilman et al. 2008); HD by clinical and genetic testing (Kieburtz et al. 1996); ET by published clinical research criteria (Louis et al. 2007); CA by genetic testing or results of neurological, neuropsychological, and magnetic resonance imaging testing; MS by the revised McDonald Criteria (Thompson et al. 2018); and ALS according to the El Escorial Criteria from the World Federation of Neurology (Brooks et al. 2000). Across all patient groups, we applied exclusion criteria: (1) a history of communication disorders unrelated to disease (e.g., problems with speech comprehension or expression), (2) a history of other neurological disorders potentially affecting speech, and (3) non-native Czech speaker.

At the time of examination, all PD patients were de-novo examined before antiparkinsonian treatment was started. Thirteen of 20 MSA patients were on stable antiparkinsonian medication, for at least 4 weeks, consisting of various doses of levodopa alone or combined with different dopamine agonists and/or amantadine, with mean L-dopa equivalent of 709 (SD 409) mg. Similarly, 10 of 18 PSP patients were on stable antiparkinsonian medication, for at least 4 weeks, consisting of various doses of levodopa alone or combined with different dopamine agonists and/or amantadine, with mean L-dopa equivalent of 790 (SD 388) mg. In MSA and PSP patients, the speech investigation was performed in best on-state after morning medication intake.

PD, ET, and MS were selected because they are one of the most prevalent neurological etiologies associated with dysarthria, CA due to its frequent association with ataxic dysarthria and HD as a good model associated with hyperkinetic dysarthria; the groups of speakers with PSP, MSA and ALS was chosen because of the availability of a relatively large number of speakers with this diagnosis and the association of this disease with different types of mixed and more severe dysarthria.

Additionally, 306 healthy subjects (158 women, 148 men) with a mean age of 59.1 (SD = 13.2, range: 31–87) years and no history of neurological or communication disorders served as controls to match the wide age and gender range of the investigated neurodegenerative diseases.

### Clinical evaluation

The disease severity of PD was assessed according to the motor score of the Movement Disorders Society–Unified Parkinson’s Disease Rating Scale (MDS-UPDRS) Part III (Goetz et al. 2008), PSP and MSA by The Natural History and Neuroprotection in Parkinson sPlus Syndromes–Parkinson Plus Scale (NNIPPS-PPS); (Payan et al. 2011), HD by the motor score of the Unified Huntington’s Disease Rating Scale (UHDRS); (Kieburtz et al. 1996), ET by the Tremor Research Group Essential Tremor Rating Assessment Scale (TETRAS); (Elble et al. 2012), CA by the Scale for the Assessment and Rating of Ataxia (SARA); (Schmitz-Hübsch et al. 2006), MS by the Expanded Disability Status Scale (EDSS); (Kurtzke 1983), and ALS by the ALS Functional Rating Scale–Revised (ALSFRS-R); (Cedarbaum et al. 1999). Disease duration was estimated based on the self-reported occurrence of the first motor symptoms.

### Speech examination

Speech recordings were performed in a quiet room with a low ambient noise level using a head-mounted condenser microphone (Beyerdynamic Opus 55) placed approximately 5 cm from the subject’s mouth. Speech signals were sampled at 48 kHz with a 16-bit resolution. Each subject was recorded during a single session using the standardized protocol guided by a speech specialist; the detailed speech protocol has been published previously and is freely accessible (Rusz et al. 2021a, b). The participants were instructed to perform the same reading passage task twice on a standardized text of 80 words.

### Auditory–perceptual estimates of dysarthria presence, type, and severity

The presence, type, and severity of dysarthria were determined by the consensus auditory–perceptual judgment of two speech-language pathologists with more than 10 years of experience in movement disorders, who were aware of each patient’s medical diagnosis. The judgment was based on offline audio recordings, including sustained vowels, fast syllable repetitions, reading passages, and monologue, following the perceptual criteria outlined by (Darley et al. 1969a, b). The dysarthria types identified across eight neurological conditions included hypokinetic, hyperkinetic, ataxic, spastic, flaccid–spastic, spastic–ataxic, hypokinetic–spastic, hypokinetic–ataxic, and hypokinetic–spastic–ataxic (Table 1). In addition, the severity of dysarthria was rated on a 4-point scale (0 = none, 1 = mild, 2 = moderate, 3 = severe). The lower average dysarthria severity with a dominant occurrence of mild dysarthria was observed only for the PD and MS groups. All patients had perceptual dysarthria.

### Long-term average spectrum

The pause intervals longer than 30 ms were determined and removed from the speech signal using an automatic segmentation tool for connected speech (Hlavnicka et al. 2017). A speech signal was then partitioned into sections of 50-ms length (50% overlap); (Kacha et al. 2020) multiplied by the Hamming window (Oppenheim and Schafer 2010). Power spectra were computed using the fast Fourier transform in each section and then averaged to obtain the LTAS estimate; the algorithm is known as the Welch’s method (Hayes 1996). Subsequently, the four spectral moments were derived (Miller & Rochwarg, 1970). The first spectral moment represents the *spectral mean*, a measure of the concentration of the frequency distribution of energy. The second spectral moment represents the *spectral standard deviation*, a measure of the variation of the frequency distribution of energy. The third spectral moment represents s*pectral skewness*, a measure of the asymmetry of the frequency distribution of energy; negative skewness means that the left tail of energy distribution is longer and vice versa. The fourth spectral moment represented by the *spectral kurtosis*, a measure of outliers in the energy distribution. The comprehensive algorithmic details were published previously (Svihlik et al. 2023). The spectral moment values were calculated as the average of the moments from two realizations of the reading passage task to provide greater speech assessment stability (Rusz et al. 2021a, b).

### Statistical analysis

One-way analysis of covariance with post hoc Fisher’s least significant difference test was applied to evaluate group differences. All analyses were controlled for age, sex and disease duration (covariates); intergroup differences among diseases and dysarthria types were, in addition, controlled for dysarthria severity. Due to the exploratory nature of the analyses, the main findings are reported without adjustment, with a two-tailed p-value < 0.05 considered statistically significant. In addition, results adjusted using the Bonferroni correction are also presented for comparison. Pearson partial correlation analyses, controlling for age and sex were performed to assess associations between speech variables and motor severity scales.

## Results

### Effect of neurological disease type

**Fig. 1:**
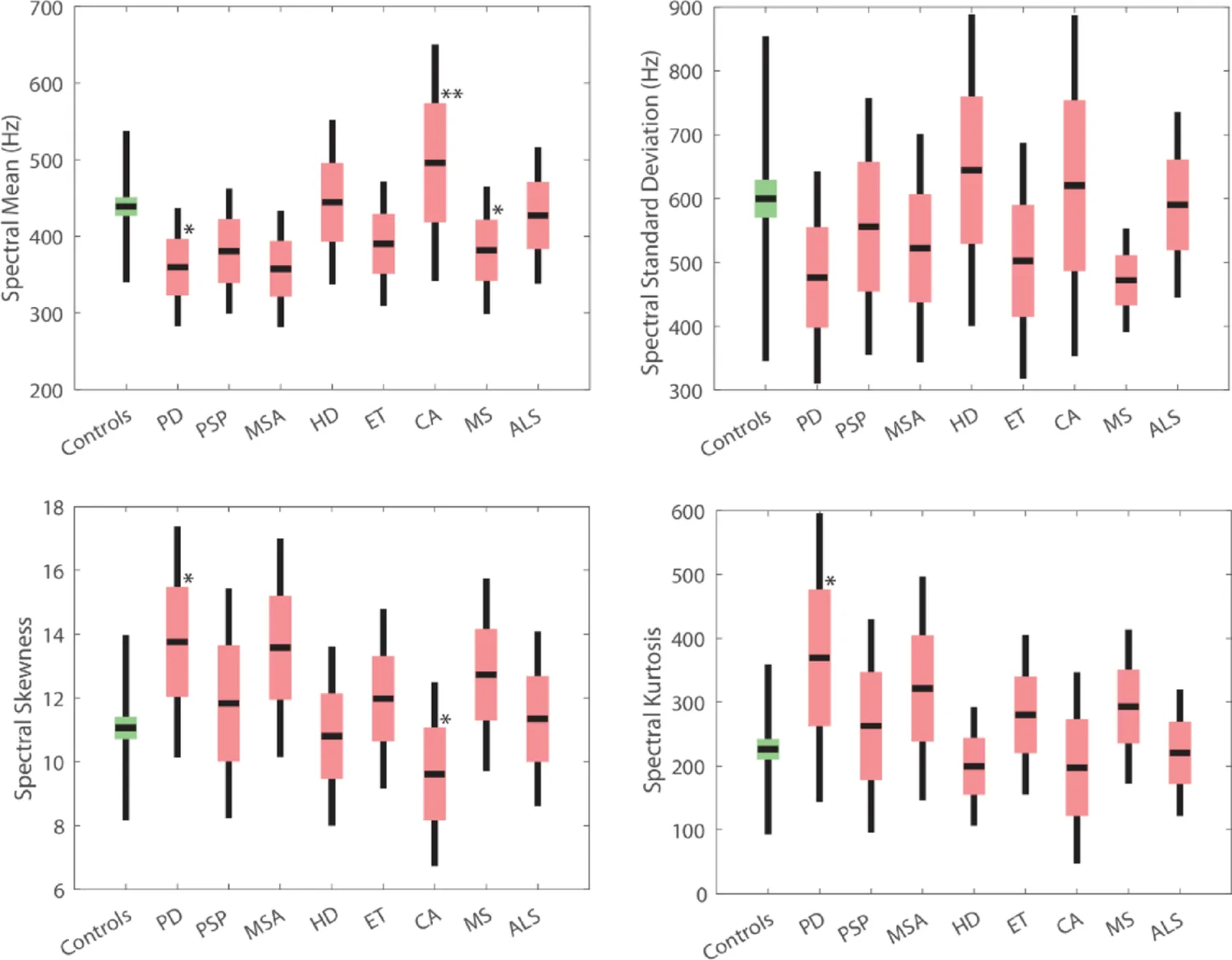
Results of spectral moments in reading passage among the different neurodegenerative disease types compared to healthy controls adjusted by age, sex, disease duration and dysarthria severity with ***, **, * referring to *p* < .001, *p* < .01, and *p* < .05, respectively. Horizontal lines represent the means, boxes represent 95% confidence interval, and whiskers represent the standard deviation. PD = Parkinson’s disease; PSP = progressive supranuclear palsy; MSA = multiple system atrophy; HD = Huntington’s disease; ET = essential tremor; CA = cerebellar ataxia; MS = multiple sclerosis; ALS = amyotrophic lateral sclerosis

Compared to controls (Fig. 1), PD showed lower spectral mean (*p* = .019), higher spectral skewness (*p* = .017), and higher spectral kurtosis (*p* = .01). Conversely, for CA, the spectral mean was higher (*p* = .008) and spectral skewness lower (*p* = .032). MS demonstrated only a lower spectral mean (*p* = .04). Considering differences between individual patient groups (Figure S1-S3), the significant differences can be mainly seen for CA and HD versus other neurodegenerative diseases such as MSA among spectral mean, spectral skewness, and spectral kurtosis. Interestingly, spectral skewness was significantly different among parkinsonian groups (Figure S2).

### Effect of dysarthria type

**Fig. 2:**
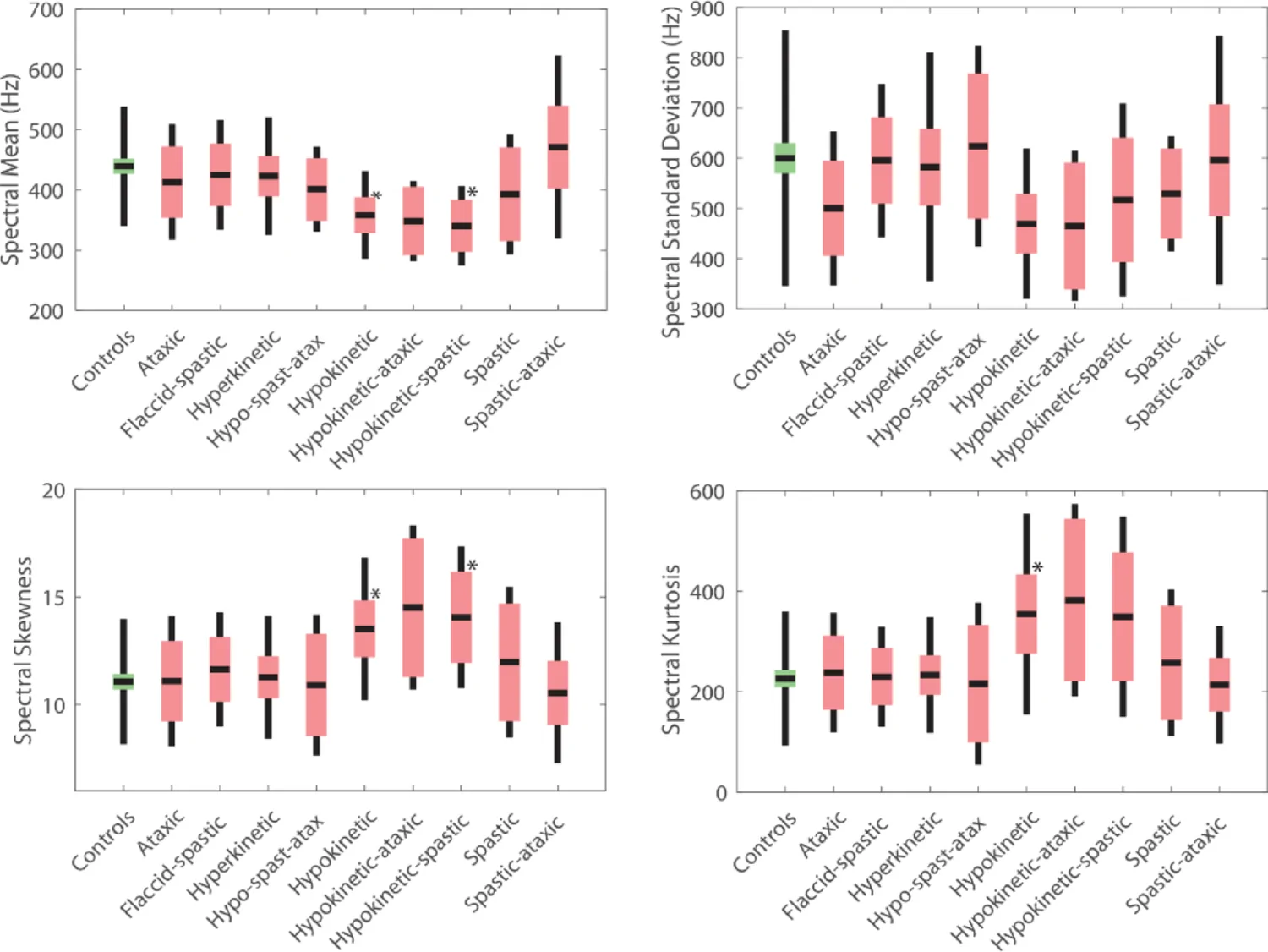
Results of spectral moments in reading passage among the different dysarthria types compared to healthy controls adjusted by age, sex, disease duration and dysarthria severity with ***, **, * referring to *p* < .001, *p* < .01, and *p* < .05, respectively. Horizontal lines represent the means, boxes represent 95% confidence interval, and whiskers represent the standard deviation

In general, compared to controls (Fig. 2), hypokinetic dysarthria and mixed dysarthria with hypokinetic elements tend to show lower spectral mean, higher spectral skewness, and higher spectral kurtosis. Across individual dysarthria types, the observed results followed patterns comparable to those seen in controls (Figures S4–S6). Based on spectral mean, spectral kurtosis, and spectral skewness, there were significant differences between dysarthrias with hypokinetic elements and dysarthrias with spastic, ataxic, and hyperkinetic elements, which were not significantly different from healthy controls.

### Effect of dysarthria severity

**Fig. 3:**
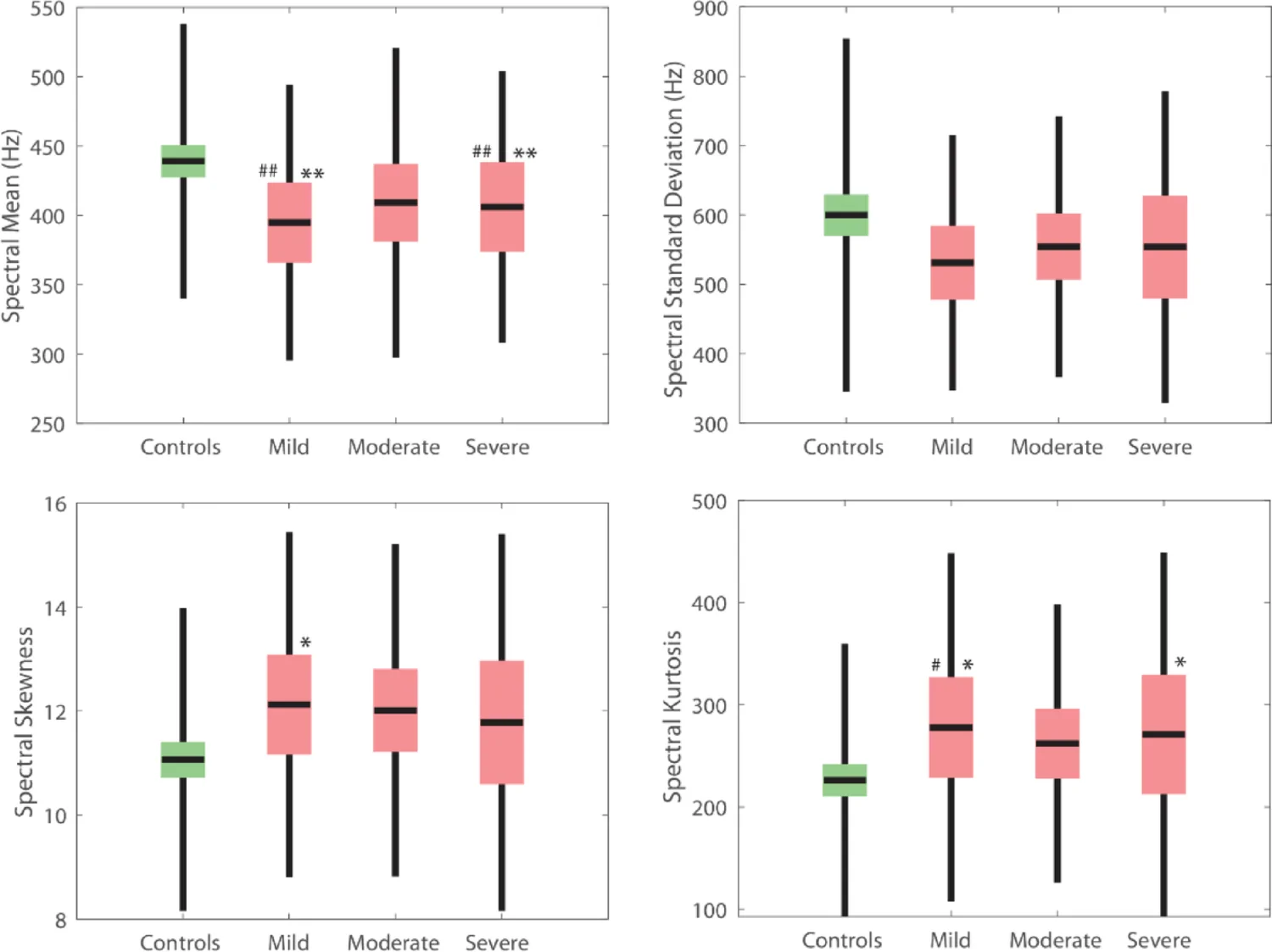
Results of spectral moments in reading passage among the different dysarthria severities compared to healthy controls adjusted by age, sex, and disease duration with ***, **, * referring to *p* < .001, *p* < .01, and *p* < .05, respectively, and ##, # referring to *p* < .01 and *p* < .05 after Bonferroni adjustment, respectively. Horizontal lines represent the means, boxes represent 95% confidence interval, and whiskers represent the standard deviation

In general, compared to controls (Fig. 3), spectral mean and spectral standard deviation were reduced, while spectral skewness and spectral kurtosis were elevated across all dysarthria severities. There were no differences in spectral moments across dysarthria severities.

### Relation between LTAS measures and disease severity scales

The significant relationships were found for PD group, where MDS-UPDRS III scale was correlated to all four moments including spectral mean (*r* = -.55, *p* = .02), spectral standard deviation (*r* = -.67, *p* = .002), spectral skewness (*r* = .62, *p* = .006), and spectral kurtosis (*r* = .69, *p* = .002). No other significant correlations were found (Table S1).

## Discussion

This study is the first to demonstrate LTAS speech analysis in a large cohort of 461 speakers, including controls and patients with various neurological diseases and different types and severity of dysarthria. We observed divergent patterns of LTAS features across PD, CA, and MS independent of dysarthria severity. The hypokinetic dysarthria types were mainly affected. The extent of dysarthria was not a major factor in the observed changes in LTAS. The spectral mean and spectral skewness primarily captured significant differences between disease and dysarthria types. Nevertheless, changes in spectral standard deviation followed a pattern similar to that of the spectral mean, while changes in spectral kurtosis and spectral skewness occurred in the opposite direction.

### Effect of neurological disease type

Compared with controls, the spectral mean tended to be lower in PD and MS, while elevated in CA. These observed trends in spectral moment are in good agreement with previous literature on LTAS in PD (Dromey 2003; Kacha et al. 2020; Svihlik et al. 2022; Tjaden et al. 2010) and MS (Tjaden et al. 2010). Physiologically, a previous study in PD demonstrated that LTAS changes were mainly correlated with voice quality, articulatory precision, articulation rate, and fundamental frequency (Svihlik et al. 2023). Decreased voice quality and imprecise articulation have been documented in PD, CA, and MS (Casper et al. 2007), whereas articulation rate tends to be consistently lower across CA and MS (Rusz et al. 2024). Therefore, we may hypothesize that the difference between lower spectral mean in PD and MS and the elevated spectral mean in CA is mainly due to changes in fundamental frequency. While PD and MS have been shown to manifest monopitch (Rusz et al. 2024), the speech in CA is typically excessive, leading to increased F0 variability (Cloud et al. 2024). From a clinical point of view, this finding might have important implications as the differentiation of the idiopathic late-onset CA from cerebellar variant of MSA early in the disease course remains a major diagnostic challenge (Ham et al. 2025; Lin et al. 2016) Also, since LTAS features appear to be relatively unchanged in PSP compared to controls, another potential diagnostic clinical usefulness in speech screening via LTAS can be for differentiation between PD and PSP as well as MSA and PSP (Daoudi et al. 2022). Last but not least, we found relationship between LTAS features and motor score of the MDS-UPDRS in PD cohort, which could potentially indicate speech acoustic parameters for use in a non-invasive screening tool for other motor impairment in PD patients (Madetko-Alster et al. 2025).

### Effect of dysarthria type

Previous research identified the usefulness of LTAS mainly in patients with PD and hypokinetic dysarthria with applications such as tracking effects of stimulation-induced speech worsening due to deep brain stimulation (Tripoliti et al. 2011), monitoring early disease progression for neuroprotective clinical trials (Simek et al. 2025), and indexing changes in the hypokinetic and hyperkinetic symptoms due to acute effects of levodopa (Sousa et al. 2025). Although LTAS has been investigated only rarely in dysarthria types other than hypokinetic dysarthria (Tjaden et al. 2010), the present findings do not support its usefulness across other dysarthria types. Indeed, we found significantly altered LTAS features only in hypokinetic dysarthria and mixed dysarthrias that manifest with hypokinetic elements. Perceptually, reduced LTAS mean values in hypokinetic dysarthria may reflect weakened upper harmonics, particularly those associated with consonants, especially fricatives. At the same time, acoustic energy is predominantly concentrated in lower frequencies, mainly corresponding to vowels. As hypokinetic dysarthria severity increases, speech may further deteriorate due to factors such as rebound tremor or heightened rigidity and hypokinesia. Consequently, lower LTAS mean values in hypokinetic speech likely reflect breathy phonation and reduced articulatory precision.

### Effect of dysarthria severity

Although there were significant differences in LTAS features between controls and patients with varying levels of dysarthria, there was no progressive increase in dysarthria severity. This is in contrast with previous literature showing the relationship between auditory-perceptual evaluation of dysarthria severity and LTAS metrics in PD (Tjaden et al. 2010). Indeed, this discrepancy between the current and previous study (Tjaden et al. 2010) could be due to differences in disease severity, as spectral moments vary considerably across diseases. Consequently, grouping severity levels across diseases likely led to the averaging of disease-specific spectral moments, obscuring progressive severity-related trends.

### Implications for differential diagnosis of dysarthria

The aim of this study was to evaluate the sensitivity of LTAS as a potential surrogate endpoint for the differential diagnosis of distinct dysarthria patterns. Although some differences observed in this study may contribute to distinguishing dysarthria types or underlying disease etiology, the findings suggest that changes in LTAS may reflect a more general marker of speech impairment, showing severity-related variation across multiple neurological conditions. Future work may benefit from combining LTAS with other speech-based measures to improve sensitivity for the differential diagnosis of dysarthria (Garima Sharma, 2020); (Garima Vyas 2016); (Sharma et al. 2022); (Shafer et al. 2005); (Garima Sharma, 2021). To the best of our knowledge, only limited research has attempted to differentiate among neurological diseases with diverse clinical manifestations using acoustic speech metrics aligned with frameworks such as the Mayo perceptual classification (Darley et al. 1969a), (Darley et al. 1969b). Previous studies provide mixed but informative evidence. For example, (Liss et al. 2009) demonstrated that rhythm metrics can distinguish dysarthric speech from healthy controls and even between dysarthria subtypes when these are well defined. Using a broader set of acoustic descriptors, (Kim et al. 2011) found that classification accuracy was generally lower for dysarthria type than for disease type or severity. (Rowe et al. 2022) applied acoustic measures within a sequential motion rate paradigm and identified distinct articulatory phenotypes across clinical groups. More recently, (Illner et al. 2023) showed that vowel imprecision represents a common feature of articulatory impairment, with severity-related variation across different dysarthria types. Nevertheless, despite these promising findings, acoustic similarity across etiologies and dysarthria types has also been reported for several measures, including speaking rate and voice onset time (Weismer 2006). Accumulating evidence suggests that diverse neuropathologies may affect the neuromotor control of speech production in similar ways, resulting in overlapping acoustic manifestations across different conditions (Kim et al. 2011).

### Limitations

This study has several limitations. Although it draws upon one of the largest multi-disease datasets to date, the analyses would benefit from larger sample sizes within each disease and dysarthria subtype. In some cases, the presence of mixed dysarthria reduced the number of participants available for analyses of specific subtypes. Among each group with specific diagnosis, there are important differences such as sex and disease duration, which might have an impact on speech performance. Although we adjusted all these potential confounding factors in statistical analysis, future research with larger and more balanced cohorts across diverse diseases and dysarthria types is warranted to validate and extend these findings. Moreover, as this study was conducted exclusively in Czech, further work is needed to assess the language independence of the applied methods. Encouragingly, recent multilingual studies have reported broadly similar dysarthria profiles across different languages in PD, HD, and ALS (Fahed et al. 2024; Kothare et al. 2024; Rusz et al. 2021a, b), suggesting potential cross-linguistic generalizability.

## Conclusion

Our findings suggest that LTAS-based speech analysis may provide useful cues to support differential diagnosis among neurological diseases with similar clinical manifestations. LTAS analysis is more informative when applied to specific neurological diseases rather than to pooled dysarthria types arising from different neurological etiologies. Future longitudinal studies should corroborate the sensitivity of LTAS features to disease progression among various neurological disorders.

## Supplementary Information

Below is the link to the electronic supplementary material.

Supplementary Material 1

## Data Availability

Individual participant data that underlie the findings of this study are available upon request to the corresponding author by qualified researchers. The speech data are not publicly available due to their containing information that could compromise the privacy of study participants.
