# Supplementary material for: Screening speech disorders in progressive neurological diseases via long-term average spectrum
Source: J Neural Transm (Vienna). 2026 May 14;133(9):2493–502. doi: 10.1007/s00702-026-03176-2 (PMC13687200; doi:10.1007/s00702-026-03176-2)
Supplement: Supplementary file 1 [file 702_2026_3176_MOESM1_ESM.pdf]

Supplementary material

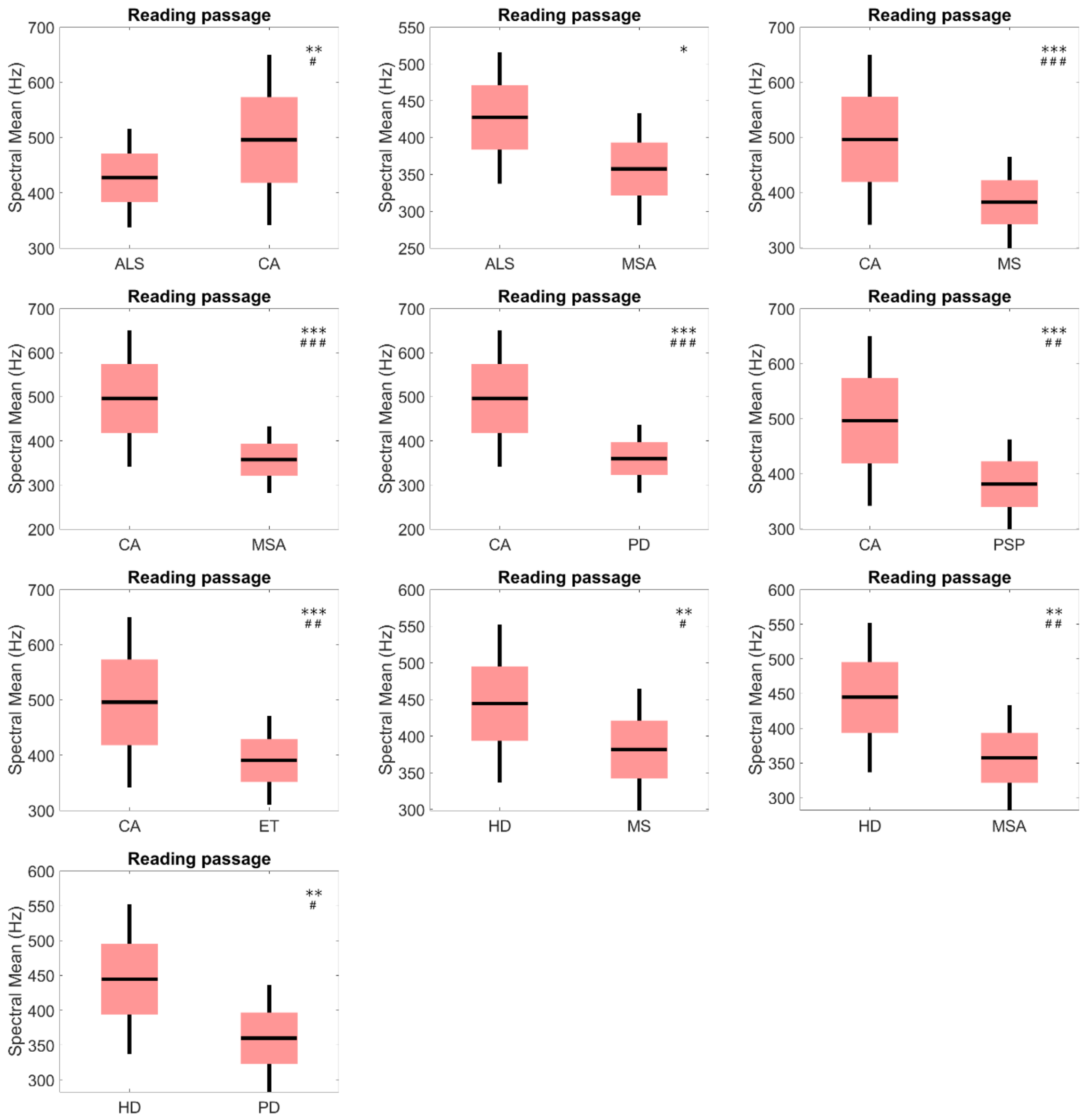

Fig. S1 Results of the spectral mean in reading passage across two pairs of neurological disease types adjusted by age, sex, disease duration, and dysarthria severity with ***, **, * referring to $p < .001$, $p < .01$, and $p < .05$, respectively, and ###, ##, # referring to $p < .001$, $p < .01$ and $p < .05$ after Bonferroni adjustment, respectively. Horizontal lines represent the means, boxes represent 95% confidence interval, and whiskers represent the standard deviation. PD = Parkinson's disease; PSP = progressive supranuclear palsy; MSA = multiple system atrophy; HD = Huntington's disease; ET = essential tremor; CA = cerebellar ataxia; MS = multiple sclerosis; ALS = amyotrophic lateral sclerosis

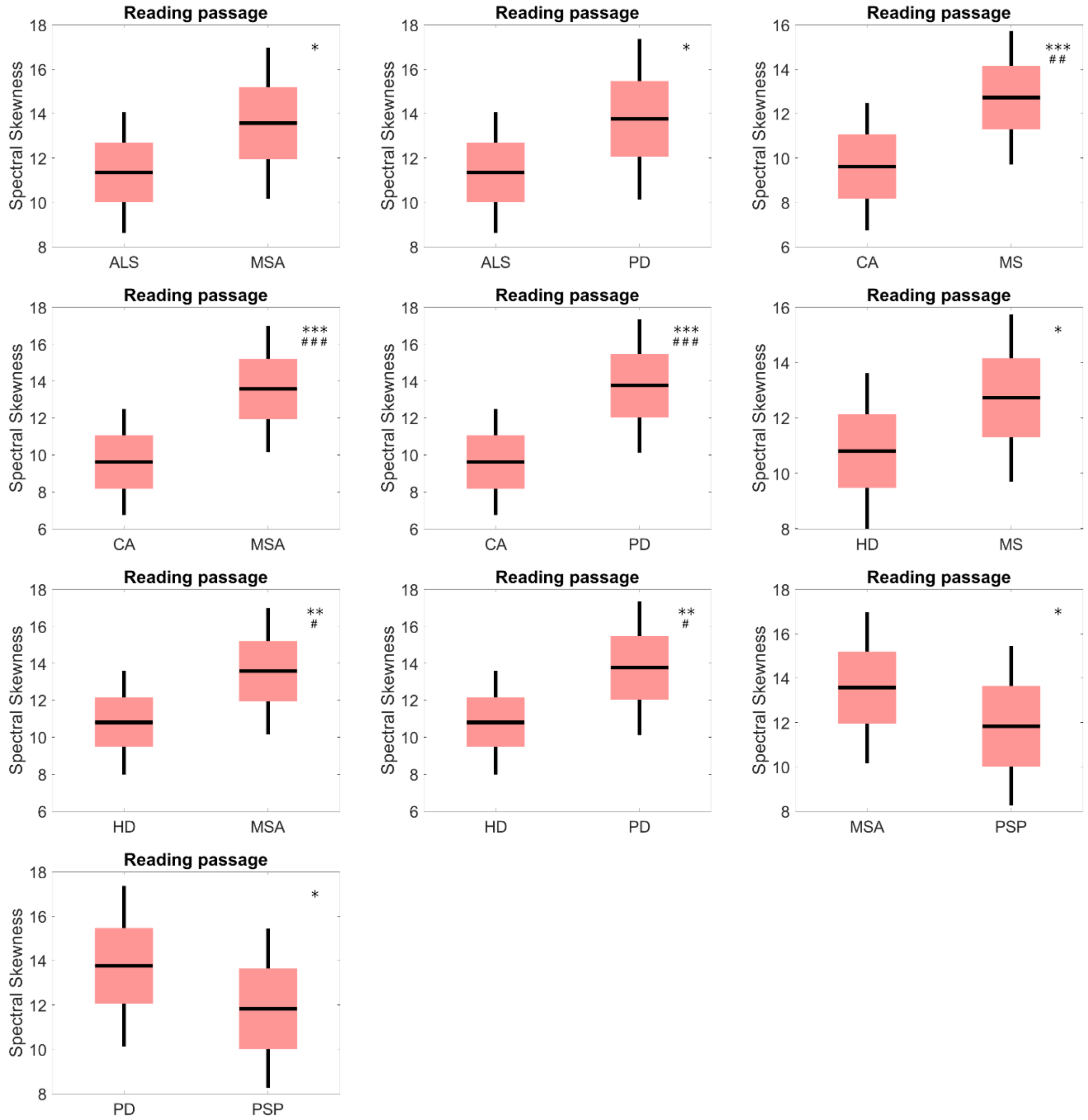

Fig. S2 Results of the spectral skewness in reading passage across two pairs of neurological disease types adjusted by age, sex, disease duration, and dysarthria severity with ***, **, * referring to p < .001, p < .01, and p < .05, respectively, and ###, ##, # referring to p < .001, p < .01 and p < .05 after Bonferroni adjustment, respectively. Horizontal lines represent the means, boxes represent 95% confidence interval, and whiskers represent the standard deviation. PD = Parkinson’s disease; PSP = progressive supranuclear palsy; MSA = multiple system atrophy; HD = Huntington’s disease; ET = essential tremor; CA = cerebellar ataxia; MS = multiple sclerosis; ALS = amyotrophic lateral sclerosis

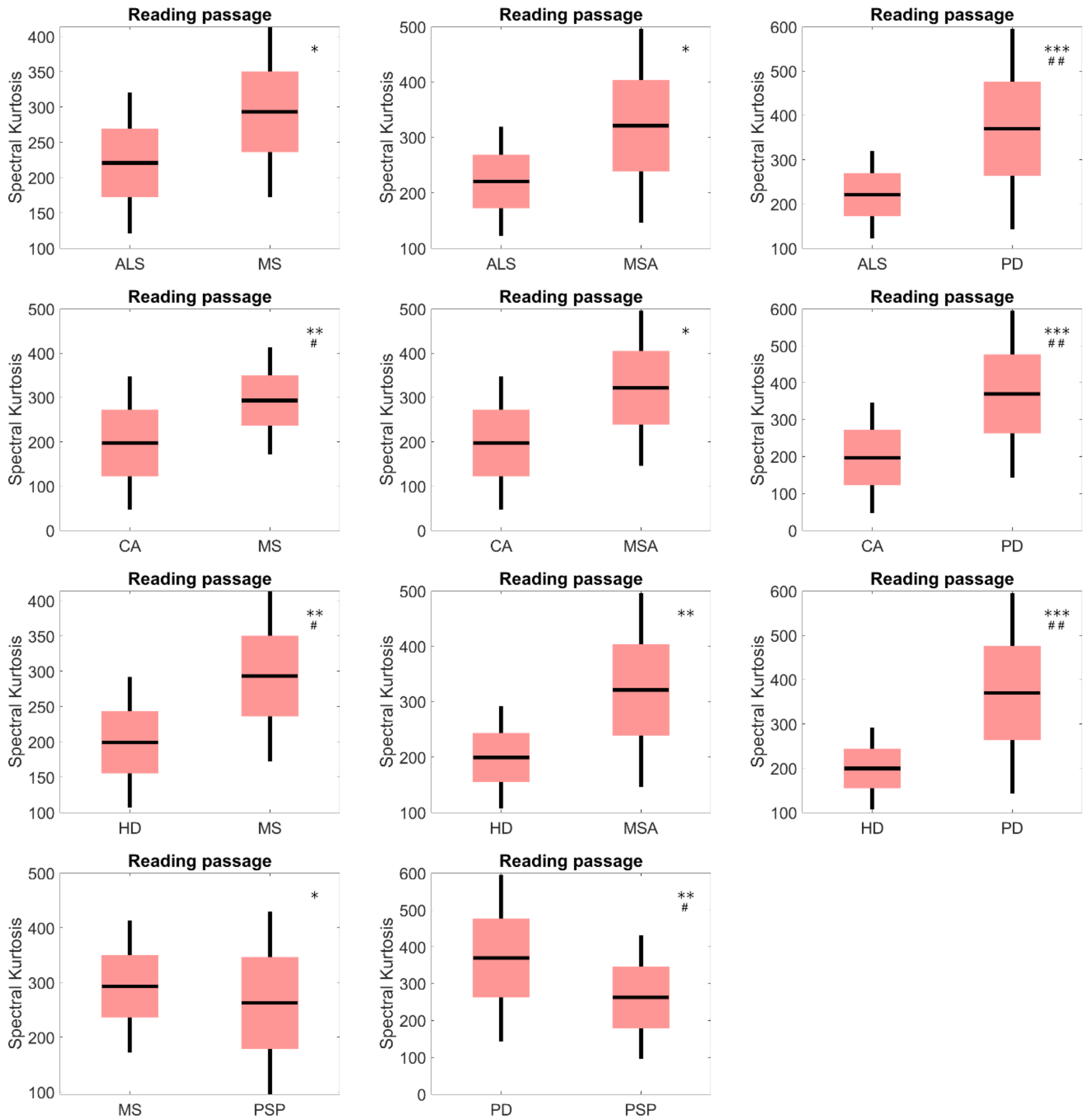

Fig. S3 Results of the spectral kurtosis in reading passage across two pairs of neurological disease **types** adjusted by age, sex, disease duration, and dysarthria severity with ***, **, * referring to $p < .001$, $p < .01$, and $p < .05$, respectively, and ###, ##, # referring to $p < .001$, $p < .01$ and $p < .05$ after Bonferroni adjustment, respectively. Horizontal lines represent the means, boxes represent 95% confidence interval, and whiskers represent the standard deviation. PD = Parkinson's disease; PSP = progressive supranuclear palsy; MSA = multiple system atrophy; HD = Huntington's disease; ET = essential tremor; CA = cerebellar ataxia; MS = multiple sclerosis; ALS = amyotro-phic lateral sclerosis

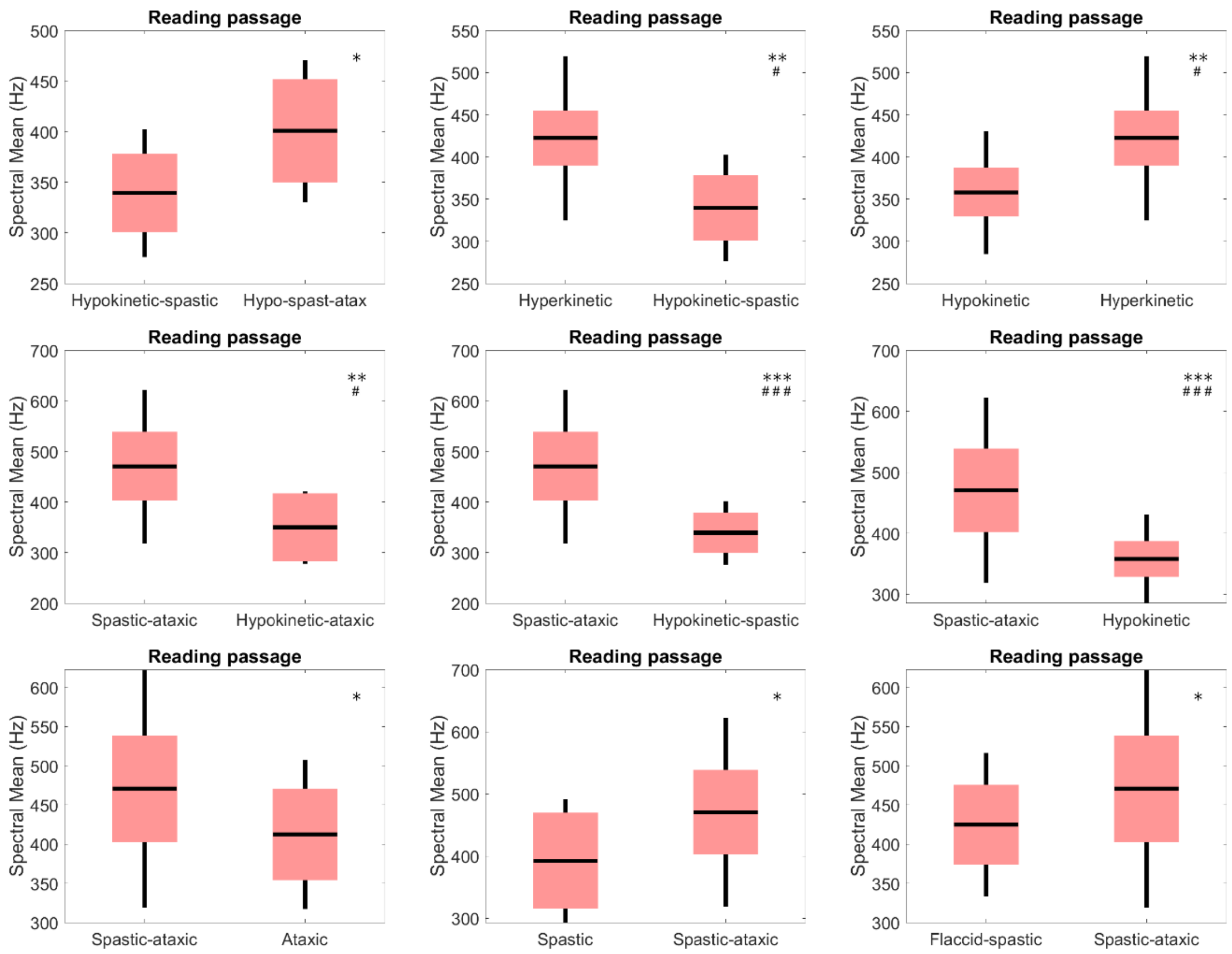

Fig. S4 Results of the spectral mean in reading passage across two pairs of dysarthria types adjusted by age, sex, disease duration, and dysarthria severity with ***, **, * referring to $p < .001$, $p < .01$, and $p < .05$, respectively, and ###, ##, # referring to $p < .001$, $p < .01$ and $p < .05$ after Bonferroni adjustment, respectively. Horizontal lines represent the means, boxes represent 95% confidence interval, and whiskers represent the standard deviation

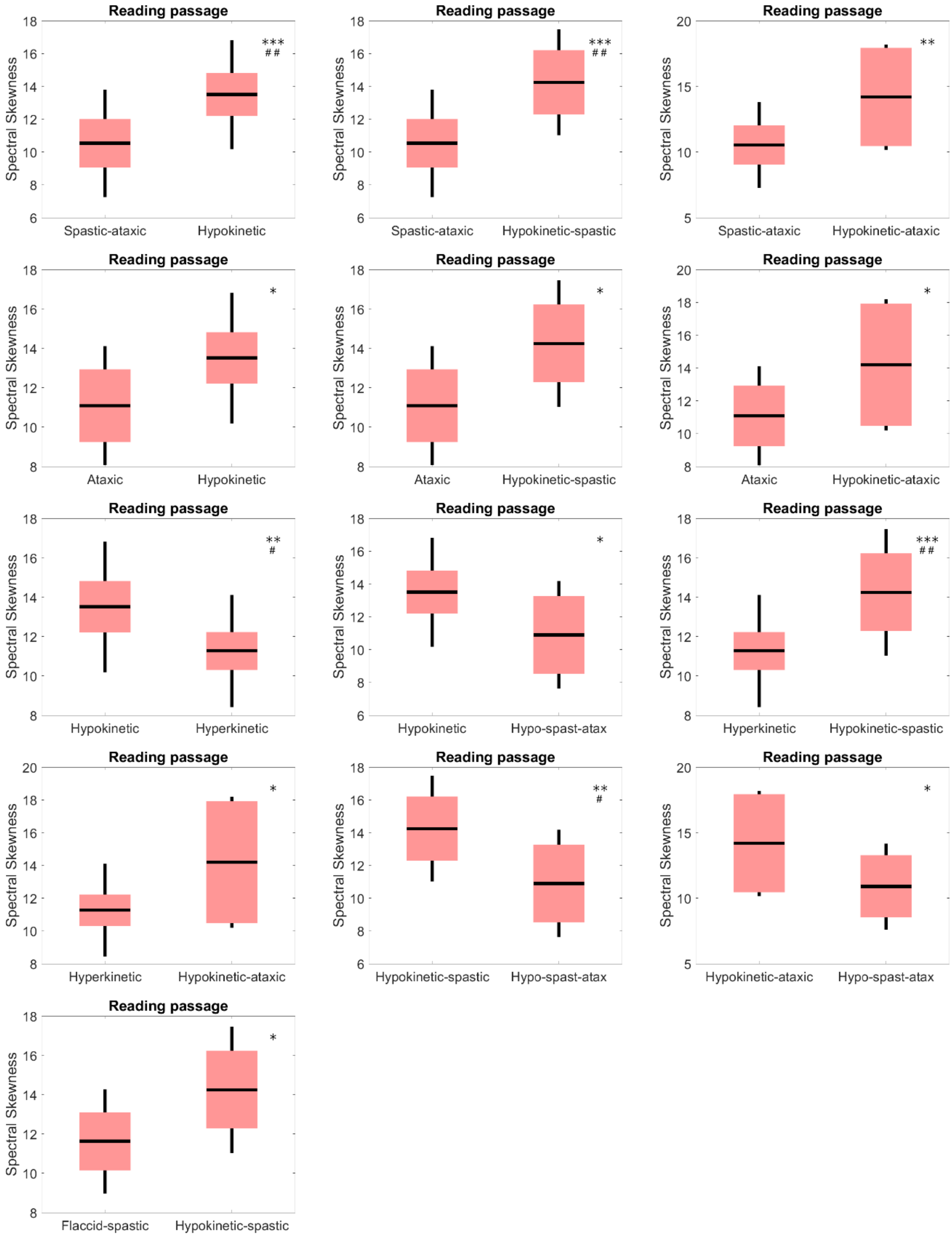

Fig. S5 Results of the spectral skewness in reading passage across two pairs of dysarthria types adjusted by age, sex, disease duration, and dysarthria severity with ***, **, * referring to $p < .001$, $p < .01$, and $p < .05$, respectively, and ###, ##, # referring to $p < .001$, $p < .01$ and $p < .05$ after Bonferroni adjustment, respectively. Horizontal lines represent the means, boxes represent 95% confidence interval, and whiskers represent the standard deviation

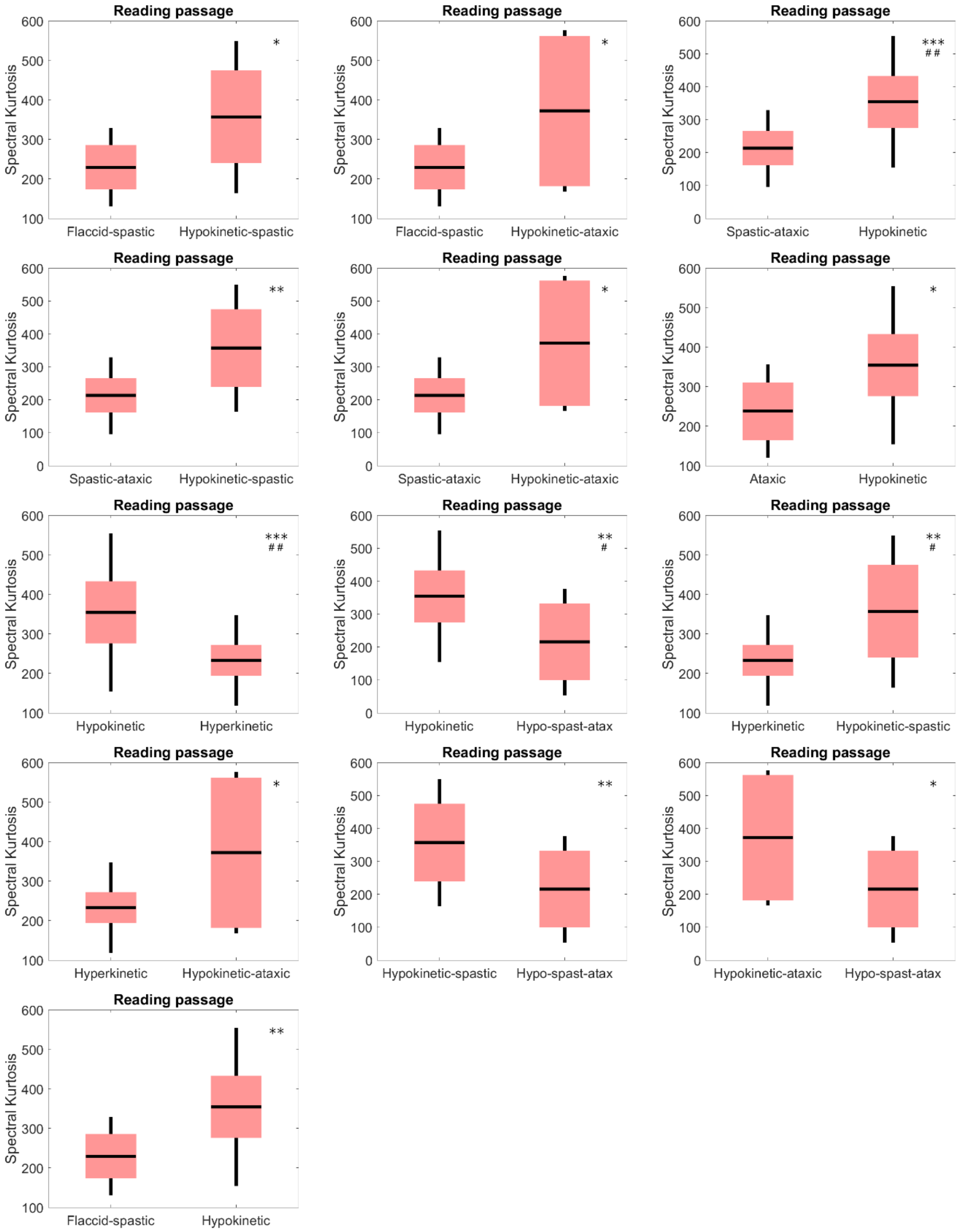

Fig. S6 Results of the spectral kurtosis in reading passage across two pairs of dysarthria types adjusted by age, sex, disease duration, and dysarthria severity with ***, **, * referring to $p < .001$, $p < .01$, and $p < .05$, respectively, and ###, ##, # referring to $p < .001$, $p < .01$ and $p < .05$ after Bonferroni adjustment, respectively. Horizontal lines represent the means, boxes represent 95% confidence interval, and whiskers represent the standard deviation

Table S1. Pearson partial correlation (controlled for sex and age) between LTAS measures and disease severity scales.

**Moment**	**Mean**		**Standard deviation**		**Skewness**		**Kurtosis**	
**Disea**	**r**	**p**	**r**	**p**	**r**	**p**	**r**	**p**
SCA	-0.1203	0.6571	-0.0851	0.7540	-0.0417	0.8782	0.0517	0.8492
ALS	-0.2740	0.2872	-0.1086	0.6782	-0.1331	0.6105	-0.0401	0.8784
PD	**-0.5501**	**0.0180**	**-0.6723**	**0.0022**	**0.6238**	**0.0057**	**0.6924**	**0.0015**
HN	-0.3524	0.1515	-0.3562	0.1468	0.2055	0.4134	0.2647	0.2885
MS	0.2918	0.2401	-0.3015	0.2240	-0.1361	0.5902	0.0132	0.9585
MSA	-0.3271	0.1852	-0.4393	0.0681	0.2853	0.2512	0.2894	0.2441
PSP	-0.3516	0.1818	-0.4602	0.0728	0.3055	0.2499	0.4211	0.1043
ET	0.2832	0.2548	0.1355	0.5920	-0.1210	0.6324	-0.1042	0.6808

Captions: Bold numbers indicate significant relationship.
